# Supplementary material for: A neuronal MAP kinase constrains growth of a Caenorhabditis elegans sensory dendrite throughout the life of the organism
Source: PLoS Genet. 2018 Jun 7;14(6):e1007435. doi: 10.1371/journal.pgen.1007435 (PMC6007932; doi:10.1371/journal.pgen.1007435)
Supplement: S1 Table — (PDF) [file pgen.1007435.s001.pdf]

Supplemental Table I. Strains

**Strains shown in Table I and Figure 1**
ynIs78[*flp-8*pro:GFP] X
mapk-15(hmn5) III*; ynIs78*[*flp-8*pro:GFP] X
sma-1(hmn6) V; *ynIs78*[*flp-8*pro:GFP] X
sma-1(hmn17) V; *ynIs78*[*flp-8*pro:GFP] X
tni-3(hmn2) V; *ynIs78*[*flp-8*pro:GFP] X
hmn13; *ynIs78*[*flp-8*pro:GFP] X
hmn11; ynIs78[*flp-8*pro:GFP] X
hmn14; *ynIs78*[*flp-8*pro:GFP] X
hmn15; *ynIs78*[*flp-8*pro:GFP] X
hmn16; *ynIs78*[*flp-8*pro:GFP] X
**Strains shown in Figure 2**
ynIs78[*flp-8*pro:GFP] X
mapk-15(hmn5) III*; ynIs78*[*flp-8*pro:GFP] X
sma-1(hmn6) V; *ynIs78*[*flp-8*pro:GFP] X
*sma-1(e934)*V; *ynIs78*[*flp-8*pro:GFP] X
dpy-1(e1) III; *ynIs78*[*flp-8*pro:GFP] X
mapk-15(hmn5) III*; ynIs78*[*flp-8*pro:GFP] X; *Ex*[WRM0615aA10(*mapk-15(+)* fosmid), *flp-8*pro:mCherry]
sma-1(hmn6) V; *ynIs78*[*flp-8*pro:GFP] X; *Ex*[WRM066dD12(*sma-1(+)* fosmid), *flp-8*pro:mCherry]
**Strains shown in Figure 3**
ynIs78[*flp-8*pro:GFP] X
mapk-15(hmn5) III*; ynIs78*[*flp-8*pro:GFP] X
sma-1(hmn6) V; *ynIs78*[*flp-8*pro:GFP] X
mapk-15(hmn5) III*; ynIs78*[*flp-8*pro:GFP] X; *Ex*[*hsp-16.41*pro:*mapk-15*, *hsp-16.2*pro:*mapk-15*, *rol-6(su1006)*
**Strains shown in Figure 4**
ynIs78[*flp-8*pro:GFP] X; *Ex*[*mapk-15*pro:NLS-mCherry]
mapk-15(hmn5) III*; ynIs78*[*flp-8*pro:GFP] X; *Ex*[WRM0615aA10(*mapk-15(+)* fosmid), *flp-8*pro:mCherry]
sma-1(hmn6) V; *ynIs78*[*flp-8*pro:GFP] X; *Ex*[WRM066dD12(*sma-1(+)* fosmid), *flp-8*pro:mCherry]
ynIs78[*flp-8*pro:GFP] X
mapk-15(hmn5) III*; ynIs78*[*flp-8*pro:GFP] X
mapk-15(hmn5) III*; ynIs78*[*flp-8*pro:GFP] X; *Ex*[*mapk-15*pro:*mapk-15*, *rol-6(su1006)*]
mapk-15(hmn5) III*; ynIs78*[*flp-8*pro:GFP] X; *Ex*[*flp-8*pro:*mapk-15*, *rol-6(su1006)*]
**Strains shown in Figure 5**
*Ex[flp-8*pro:mCherry, *flp-8*pro:superfolderGFP-MAPK-15, *rol-6(su1006)*]
ynIs78[*flp-8*pro:GFP] X
mapk-15(hmn5) III*; ynIs78*[*flp-8*pro:GFP] X

mapk-15(hmn5) III*; ynIs78*[*flp-8*pro:GFP] X; *Ex*[*mapk-15*pro:*mapk-15*, *rol-6(su1006)*]
mapk-15(hmn5) III*; ynIs78*[*flp-8*pro:GFP] X; *Ex*[*mapk-15*pro:MAPK-15(K42A), *rol-6(su1006)*]
mapk-15(hmn5) III*; ynIs78*[*flp-8*pro:GFP] X; *Ex*[*mapk-15*pro:MAPK-15(ΔLIR), *rol-6(su1006)*]
**Strains shown in Figure 6**
mapk-15(hmn5) III; *Ex*[*gcy-37*pro:GCY-35-HA-GFP-*SL2*-mCherry]
ynIs78[*flp-8*pro:GFP] X
mapk-15(hmn5) III*; ynIs78*[*flp-8*pro:GFP] X
mapk-15(hmn5) III*; ynIs78*[*flp-8*pro:GFP] X; *Ex*[*gcy-32*pro:GCY-35, *rol-6(su1006)*]
mapk-15(hmn5) III*; ynIs78*[*flp-8*pro:GFP] X; *Ex*[*gcy-32*pro:GCY-35(D473A), *rol-6(su1006)*]
tax-2(p691) I; mapk-15(hmn5) III*; ynIs78*[*flp-8*pro:GFP] X; *Ex*[*gcy-32*pro:GCY-35, *rol-6(su1006)*]
mapk-15(hmn5) III*; egl-4(ad450)* IV; *ynIs78*[*flp-8*pro:GFP] X
egl-4(ad450) IV; *ynIs78*[*flp-8*pro:GFP] X
mapk-15(hmn5) III*; Ex*[*gcy-37*pro:YC2.60, *unc-122*pro:RFP]
Ex[*gcy-37*pro:YC2.60, *unc-122*pro:RFP]
gcy-35(ok769) I; *Ex*[*gcy-37*pro::YC2.60, *unc-122*pro::RFP]
npr-1(ad609) X
npr-1(ad609) X*; mapk-15(hmn5)* III*; ynIs78*[*flp-8*pro:GFP] X
npr-1(ad609) X*; gcy-35(ok769)* I*; gcy-36(db42)* X
**Strains shown in Supplemental Figure S1**
tni-3(hmn2) V; *ynIs78*[*flp-8*pro:GFP] X
tni-3(hmn2) V; *ynIs78*[*flp-8*pro:GFP] X; *Ex*[WRM065bA04(*tni-3(+)* fosmid, *rol-6(su1006)*]
**Strains shown in Supplemental Figure S2**
ynIs78[*flp-8*pro:GFP] X
mapk-15(hmn5) III*; ynIs78*[*flp-8*pro:GFP] X; *Ex*[*hsp-16.41*pro:*mapk-15*, *hsp-16.2*pro:*mapk-15*, *rol-6(su1006)*
**Strains shown in Supplemental Figure S3**
mapk-15(hmn5) III; *Ex[flp-8*pro:mCherry, *flp-8*pro:superfolderGFP-MAPK-15, *rol-6(su1006)*]
ynIs78[*flp-8*pro:GFP] X
mapk-15(hmn5) III*; ynIs78*[*flp-8*pro:GFP] X
*Ex[flp-8*pro:mCherry, *flp-8*pro:superfolderGFP-MAPK-15, *rol-6(su1006)*]
**Strains shown in Supplemental Figure S4**
mapk-15(hmn5) III; *ynIs78*[*flp-8*pro:GFP] *lin-15(n765)* X; Ex[*glb-5*pro:*glb-5*(*Haw*)-mCherry, *lin-15(+)*]
ynIs78[*flp-8*pro:GFP] X
mapk-15(hmn5) III*; ynIs78*[*flp-8*pro:GFP] X
egl-4(n478) IV; *ynIs78*[*flp-8*pro:GFP] X
mapk-15(hmn5) III*; egl-4(n478)* IV; *ynIs78*[*flp-8*pro:GFP] X
