# Supplementary material for: A neuronal MAP kinase constrains growth of a Caenorhabditis elegans sensory dendrite throughout the life of the organism
Source: PLoS Genet. 2018 Jun 7;14(6):e1007435. doi: 10.1371/journal.pgen.1007435 (PMC6007932; doi:10.1371/journal.pgen.1007435)
Supplement: S2 Table — (PDF) [file pgen.1007435.s002.pdf]

Supplemental Table II. Mutant alleles
Substitutions are bracketed with the mutant sequence underlined.

**Allele**	**Sequence**
tni-3(hmn2)	GGCAAAGAAG[A>T]AGGGAACCGC
mapk-15(hmn5)	GCAACAAGAT[G>A]GTACAGAAGT
mapk-15(hmn51)	AAAAGGAGTG[G>A]ATATGTGGAG
sma-1(hmn6)	CAGAAAACAA[C>T]GAGGTGTCCG
sma-1(hmn17)	AAACGAGCGA[C>T]TTATTTTGGGT
