# Supplementary material for: A neuronal MAP kinase constrains growth of a Caenorhabditis elegans sensory dendrite throughout the life of the organism
Source: PLoS Genet. 2018 Jun 7;14(6):e1007435. doi: 10.1371/journal.pgen.1007435 (PMC6007932; doi:10.1371/journal.pgen.1007435)
Supplement: S1 Fig — (A) Schematic of the Troponin I TNI-3 showing effects of the mutant allele hmn2 and conserved motifs and domains. TnC, Troponin C. (B) Frequency of dendrite overgrowth was measured in tni-3 mutants bearing the wild-type fosmid. Error bars, standard error of proportion. n ≥ 15. (PDF) [file pgen.1007435.s003.pdf]

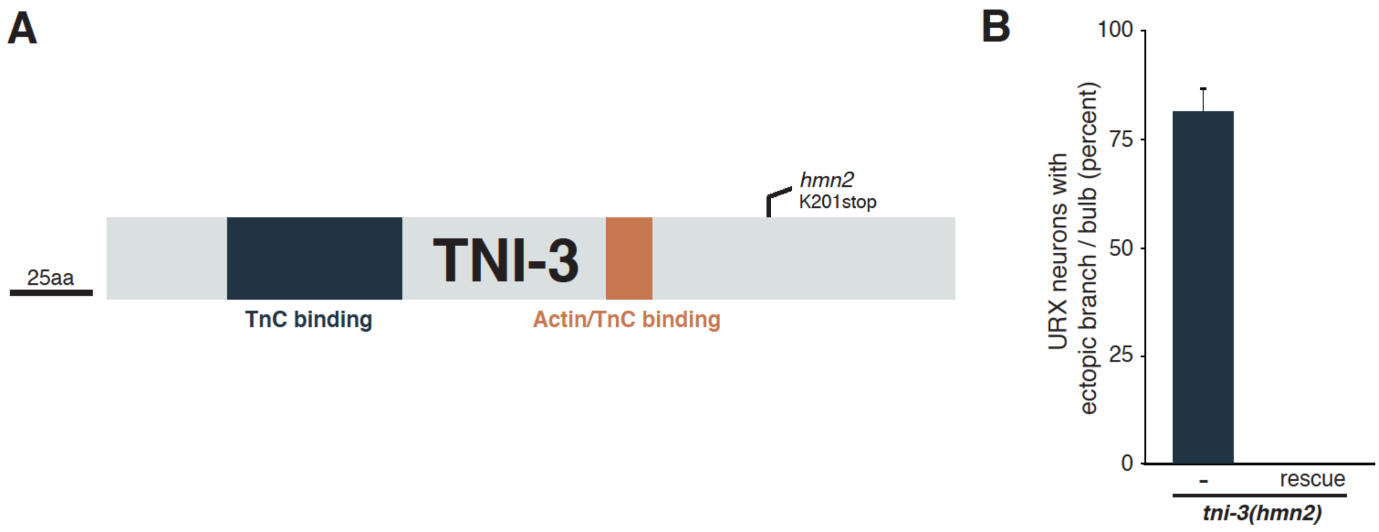

Supplemental Figure S1. Loss of TNI-3 causes ectopic branch formation

(A) Schematic of the Troponin I TNI-3 showing effects of the mutant allele *hmn2* and conserved motifs and domains. TnC, Troponin C. (B) Frequency of dendrite overgrowth was measured in *tni-3* mutants bearing the wild-type fosmid. Error bars, standard error of proportion. n ≥ 15.
