# Supplementary material for: A neuronal MAP kinase constrains growth of a Caenorhabditis elegans sensory dendrite throughout the life of the organism
Source: PLoS Genet. 2018 Jun 7;14(6):e1007435. doi: 10.1371/journal.pgen.1007435 (PMC6007932; doi:10.1371/journal.pgen.1007435)
Supplement: S2 Fig — mapk-15 mutants bearing a transgene encoding a heat-shock-inducible mapk-15(+) genomic fragment (hsp:mapk-15) were subjected at the indicated developmental stage to a brief heat shock (30 min, 34°C) or not (–), recovered, and dendrite and nose lengths were measured at the indicated stage. Embryo, mixed-stage embryos. 2Ad, second day of adulthood. 4Ad, fourth day of adulthood. p-values, Mann-Whitney U-test. Colored bars are individual animals, black bars are population averages. n ≥ 50 in all cases. (PDF) [file pgen.1007435.s004.pdf]

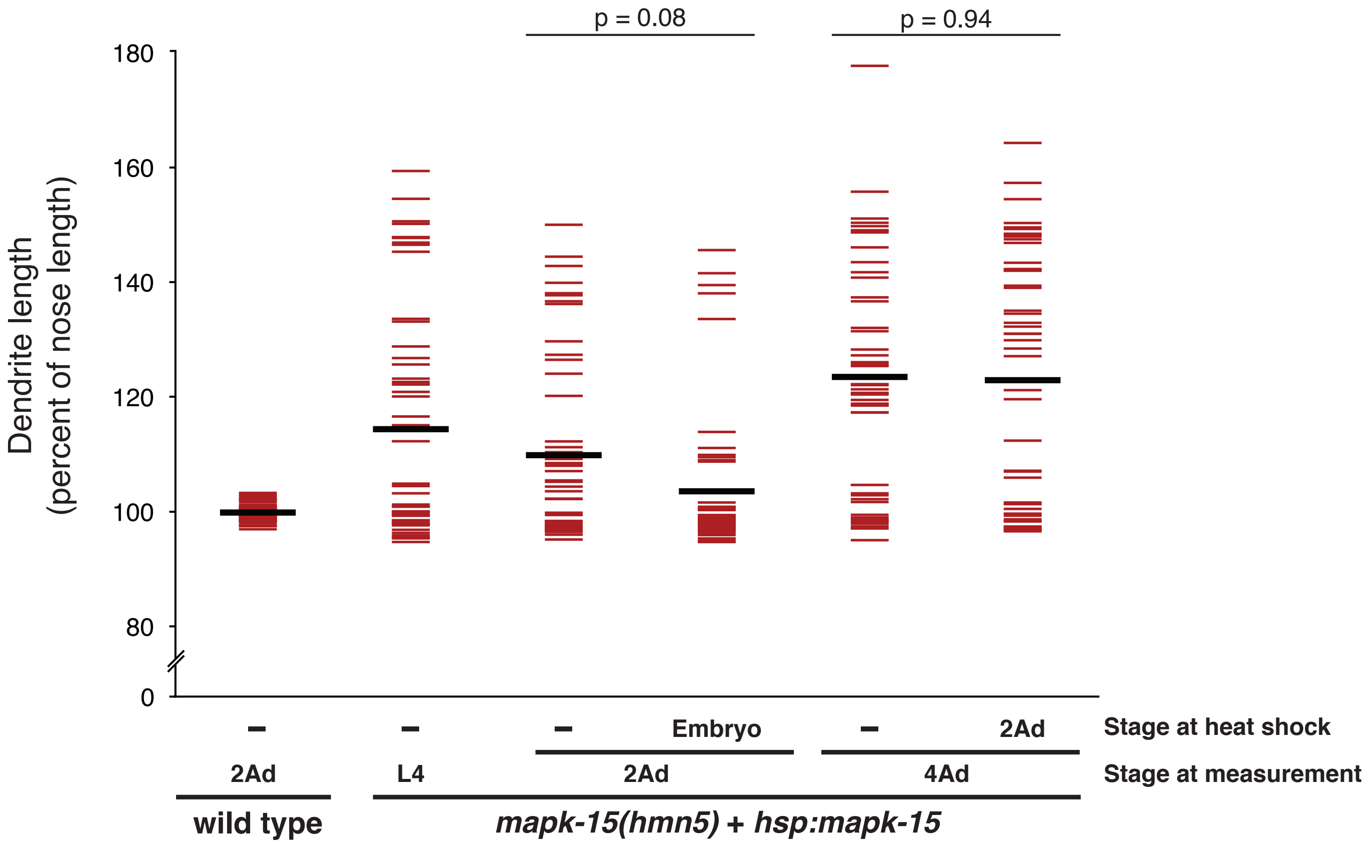

Supplemental Figure S2. Induction of MAPK-15 expression at early or late developmental timepoints does not efficiently rescue dendrite overgrowth

mapk-15 mutants bearing a transgene encoding a heat-shock-inducible *mapk-15(+)* genomic fragment *(hsp:mapk-15)* were subjected at the indicated developmental stage to a brief heat shock (30 min, 34°C) or not (–), recovered, and dendrite and nose lengths were measured at the indicated stage. Embryo, mixed-stage embryos. 2Ad, second day of adulthood. 4Ad, fourth day of adulthood. p-values, Mann-Whitney U-test. Colored bars are individual animals, black bars are population averages. $n \geq 50$ in all cases.
