# Supplementary material for: A neuronal MAP kinase constrains growth of a Caenorhabditis elegans sensory dendrite throughout the life of the organism
Source: PLoS Genet. 2018 Jun 7;14(6):e1007435. doi: 10.1371/journal.pgen.1007435 (PMC6007932; doi:10.1371/journal.pgen.1007435)
Supplement: S3 Fig — (A) Wild-type or mapk-15 animals with or without flp-8pro:superfolderGFP-MAPK-15 were synchronized as two-day adults and dendrite and nose lengths were measured. Colored bars, individual animals; black bars, population averages. n = 50 in all cases. (B) mapk-15 mutant animal expressing flp-8pro:mCherry (URX) and flp-8pro:superfolderGFP-MAPK-15 (URX). Arrow, dendrite ending. (C) Schematic showing quantification of superfolderGFP-MAPK-15 enrichment at dendrite ending. Wild-type animals expressing flp-8pro:mCherry (URX) and flp-8pro:superfolderGFP-MAPK-15 (URX) were imaged. Fluorescence intensity of a 2-μm diameter circle (yellow) in a single optical plane at the dendrite ending (i, ii) or middle (iii, iv) was calculated and background fluorescence was subtracted to yield a corrected intensity (yellow numbers). Tip enrichment was calculated as the ratio of corrected intensities at the tip vs middle (i ÷ iii, ii ÷ iv). Relative enrichment of the GFP signal was calculated by first normalizing to mCherry (i ÷ ii, iii ÷ iv) to correct for local differences in cell volume, and then calculating the ratio of these normalized values ((i ÷ ii) ÷ (iii ÷ iv)). (D) Tip enrichment of superfolderGFP-MAPK-15 and mCherry. Mean ± SD: GFP, 6.3 ± 3.9, mCherry, 1.7 ± 0.7. n = 20. Colored bars, individual dendrites. (PDF) [file pgen.1007435.s005.pdf]

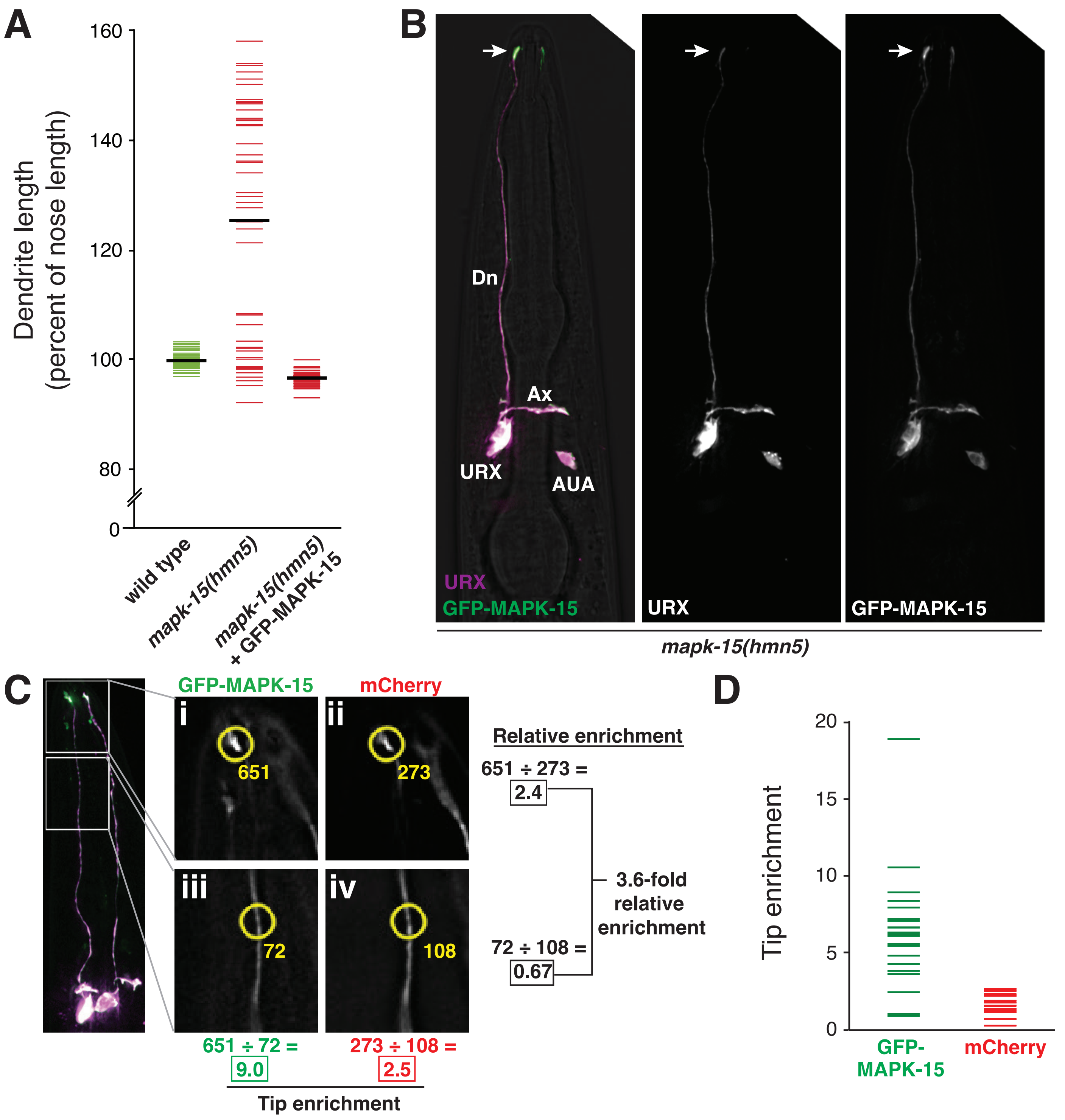

Supplemental Figure S3. A functional GFP-MAPK-15 is enriched at the dendrite ending

(A) Wild-type or *mapk-15* animals with or without *flp-8*pro:superfolderGFP-MAPK-15 were synchronized as two-day adults and dendrite and nose lengths were measured. Colored bars, individual animals; black bars, population averages. n = 50 in all cases. (B) *mapk-15* mutant animal expressing *flp-8*pro:mCherry (URX) and

*flp-8*pro:superfolderGFP-MAPK-15 (URX). Arrow, dendrite ending. (C) Schematic showing quantification of superfolderGFP-MAPK-15 enrichment at dendrite ending. Wild-type animals expressing *flp-8*pro:mCherry (URX) and *flp-8*pro:superfolderGFP-MAPK-15 (URX) were imaged. Fluorescence intensity of a 2-µm diameter circle (yellow) in a single optical plane at the dendrite ending (i, ii) or middle (iii, iv) was calculated and background fluorescence was subtracted to yield a corrected intensity (yellow numbers). Tip enrichment was calculated as the ratio of corrected intensities at the tip vs middle (i ÷ iii, ii ÷ iv). Relative enrichment of the GFP signal was calculated by first normalizing to mCherry (i ÷ ii, iii ÷ iv) to correct for local differences in cell volume, and then calculating the ratio of these normalized values ((i ÷ ii) ÷ (iii ÷ iv)). (D) Tip enrichment of superfolderGFP-MAPK-15 and mCherry. Mean ± SD: GFP, 6.3 ± 3.9, mCherry, 1.7 ± 0.7. n = 20. Colored bars, individual dendrites.
