# Supplementary material for: A neuronal MAP kinase constrains growth of a Caenorhabditis elegans sensory dendrite throughout the life of the organism
Source: PLoS Genet. 2018 Jun 7;14(6):e1007435. doi: 10.1371/journal.pgen.1007435 (PMC6007932; doi:10.1371/journal.pgen.1007435)
Supplement: S4 Fig — (A) mapk-15(hmn5) animal expressing flp-8pro:GFP (URX) and glb-5pro:glb-5-mCherry. The glb-5 construct is expressed in other neurons and diffusely in the pharynx (blue dashed outline). Boxes show magnifications of regions from the URX overgrowth (arrowheads) and dendrite middle (arrows). (B) Wild-type, mapk-15, egl-4(lf), and mapk-15;egl-4(lf) animals were synchronized as two-day adults and dendrite and nose lengths were measured. Colored bars, individual dendrites; black bars, population averages. n>33 for each genotype. (PDF) [file pgen.1007435.s006.pdf]

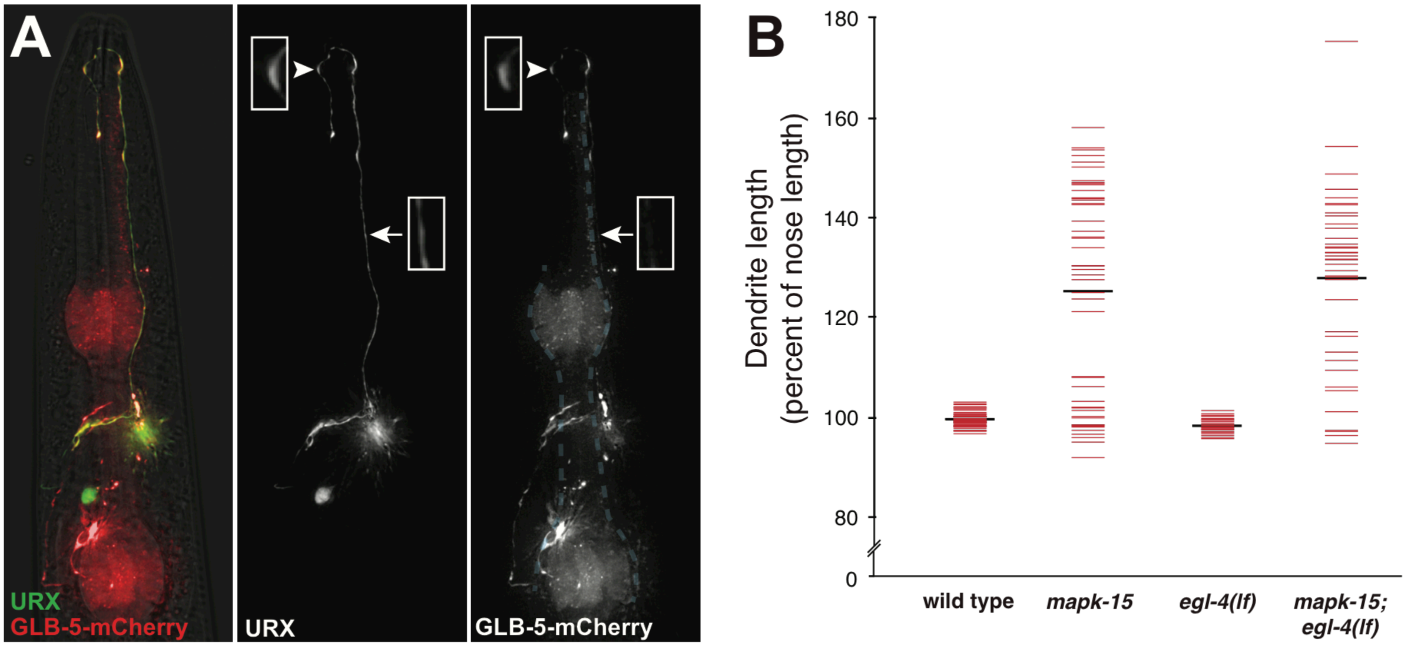

Supplemental Figure S4. The dendritic overgrowth includes an additional sensory signaling protein and is not affected by *egl-4(lf)*

(A) *mapk-15(hmn5)* animal expressing *flp-8*pro:GFP (URX) and *glb-5*pro:*glb-5*-mCherry. The *glb-5* construct is expressed in other neurons and diffusely in the pharynx (blue dashed outline). Boxes show magnifications of regions from the URX overgrowth (arrowheads) and dendrite middle (arrows). (B) Wild-type, *mapk-15*, *egl-4(lf),* and *mapk-15;egl-4(lf)* animals were synchronized as two-day adults and dendrite and nose lengths were measured. Colored bars, individual dendrites; black bars, population averages. n>33 for each genotype.
